# S100A11 as an immune-related exosomal driver of colorectal cancer progression: a novel diagnostic biomarker

**DOI:** 10.3389/fonc.2025.1590128

**Published:** 2025-06-11

**Authors:** Haibo Wang, Rongchao He, Deng Liu, Jun He, Zhong Shen

**Affiliations:** ^1^ Department of Colorectal Surgery, Hangzhou Third People’s Hospital, Hangzhou, Zhejiang, China; ^2^ Zhejiang University School of Medicine, Hangzhou, Zhejiang, China; ^3^ Department of Anesthesiology, The First Affiliated Hospital of Anhui Medical University, Hefei, China

**Keywords:** colorectal cancer, exosome, S100A11, biomarker, immune microenvironment, immunotherapy, machine learning

## Abstract

**Background:**

Colorectal cancer (CRC) is a major cause of global cancer deaths, with increasing incidence among younger populations. Despite advancements in diagnostic and therapeutic strategies, early detection and effective treatment remain major challenges. Exosomes act as critical intercellular messengers, promoting cancer growth, immune escape, and chemotherapy resistance. This study aims to identify exosome-related biomarkers in CRC and elucidate the functional significance of S100A11 in tumor progression and immune regulation.

**Methods:**

We integrated multi-cohort transcriptomic data from TCGA and GEO databases and applied a machine learning triad (LASSO-SVM-Random Forest) to identify robust exosomal biomarkers for CRC. Functional enrichment analysis, immune infiltration evaluation, and molecular docking were performed, along with *in vitro* and *in vivo* experiments, including qPCR, Western blot, IHC, apoptosis assays, and xenograft models, were performed to validate the oncogenic and immunoregulatory role of S100A11.

**Results:**

A five-gene exosome-based diagnostic panel (S100A11, CA4, PDCD4, GSTM2, SORD) was established, demonstrating excellent predictive accuracy (AUC=0.965). S100A11 was identified as a master regulator of CRC proliferation, immune modulation, and chemoresistance. Knockdown of S100A11 significantly suppressed CRC cell proliferation, induced apoptosis, and restrained tumor development in a xenograft model. Moreover, S100A11 was associated with an immunosuppressive tumor microenvironment. Pharmacogenomic analysis revealed its potential as a therapeutic target, with high binding affinity to diallyl trisulfide, suggesting novel treatment avenues.

**Conclusion:**

S100A11 mechanistically promotes CRC progression by activating oncogenic signaling and reshaping the immune microenvironment, positioning it as a clinically relevant dual-function biomarker. The integration of bioinformatics, machine learning, and experimental validation underscores the potential of exosome-derived markers for immunotherapy and precision oncology. Future studies should focus on clinical validation and the development of exosome-based immune-targeted therapies for CRC management.

## 1 Introduction

Colorectal cancer (CRC) remains the third most common malignancy worldwide, with more than 1.9 million new cases and 900,000 deaths annually (1–3). And progressively increasing prevalence in young people (4, 5). Epidemiological data demonstrate a 65% five-year relative survival probability for diagnosed individuals, reflecting current clinical outcomes (6). Despite advances in screening modalities such as colonoscopy and fecal immunochemical testing, about 25% of patients are diagnosed at a metastatic stage (7), and early-stage colon cancer is associated with favorable outcomes when treated with surgery alone or combined with neoadjuvant chemotherapy (8, 9). Evidence suggests that early detection of colon cancer leads to improvements in morbidity and mortality (10), and that late-stage metastasis makes the treatment of colon cancer more difficult (11); therefore, screening for colon cancer remains the most important part of the prevention of CRC. Screening for colon cancer remains key to cancer prevention (12), suggesting an urgent need for novel biomarkers to enable early detection and personalized treatment strategies (13, 14).

Exosomes are membrane-fused vesicles originating from the plasma membrane, with a diameter size of about tens of nanometers, with functions such as secretion, protein sorting, recycling, storage, transport and release (15–17), which can alter the tumor microenvironment, growth and progression (18). Emerging evidence suggests that tumor-derived exosomes play a role in intercellular communication, immune regulation, promotion of epithelial-mesenchymal transition and facilitation of angiogenesis and pre-metastatic ecological niche formation (19–24), which are key mediators of carcinogenesis. These nanovesicles carry specific molecular carriers, including proteins, miRNAs and mRNAs, which mirror the characteristics of the parental tumor, making them promising liquid biopsy targets (25). Current studies have identified several exosomal biomarkers associated with CRC, including mesenchymal stem cell (MSC)-derived exosomes and microRNA-17-containing vesicles (26, 27). Although existing methods for CRC screening and treatment have made very significant progress (28), there are obvious technical limitations in terms of specificity and sensitivity for early detection (29, 30), which highlights the need for multi-analyte diagnostic panels. Recent advances in machine learning have provided unprecedented opportunities to decode complex histological datasets, and integrated bioinformatics approaches have successfully identified diagnostic features for breast cancer and lung cancer (31, 32). However, methods to characterize exosome-related genes in CRC through multi-algorithmic feature selection systems remain to be explored.

The S100 family of calcium-binding proteins, especially S100A11, has attracted attention for its dual role in cancer progression by binding calcium ions and undergoing conformational changes.S100A11 is highly expressed in pancreatic cancer, where it promotes the growth of intrahepatic cholangiocarcinoma cells and facilitates melanoma metastasis (33–35). Although its functional significance in CRC has been reported, its gene function and its detailed Although its functional significance in CRC has been reported, its gene function and its detailed mechanism have rarely been studied in depth. Therefore, it is necessary to comprehensively analyze the S100A11 genome, transcriptome and functional validation.

This study fills this critical gap through three innovative dimensions: first, we employ a machine learning triad (LASSO-SVM-Random Forest) to identify robust exosome biomarkers from multi-cohort transcriptomic data, overcoming the bias of a single algorithm. Second, we elucidated the immunoregulatory role of exosome genes in shaping the CRC microenvironmental landscape using a deconvolution algorithm. Third, by combining orthogonal validation of molecular docking and functional assays, we resolved existing controversies regarding the role of S100A11 in CRC progression. Our results establish a five-gene exosome signature with excellent diagnostic performance (AUC=0.965) and reveal S100A11 as a master regulator of CRC proliferation, immune evasion, and chemoresistance, providing a mechanistic basis for the development of targeted therapies.

## 2 Materials and methods

### 2.1 Bioinformatics analysis

#### 2.1.1 Data collection and preprocessing

Data Sources: Publicly available colorectal cancer (CRC) transcriptomic datasets, including TCGA-COAD and GEO datasets (accession numbers: GSE10950, GSE23878, GSE41328, GSE44861), were systematically acquired through the Gene Expression Omnibus and The Cancer Genome Atlas portals. Exosome-related gene lists were obtained from the GeneCards database (https://www.genecards.org/) using search terms such as “exosome” and filtering for experimentally validated genes.

Batch Effect Correction: Principal Component Analysis (PCA) was performed using the ggplot2 R package to assess batch effects across datasets. The ComBat algorithm was applied for batch correction.

### 2.2 Bioinformatic mining of transcriptional disparities

Transcriptional disparities in tumor-normal pairs were delineated through empirical Bayes moderated t-tests implemented in the limma framework, with differential expression thresholds set at |log2FC|>1 and FDR<0.05.

Machine Learning-Based Feature Selection:

To prioritize diagnostic biomarkers, we implemented a multi-algorithm approach:

LASSO Regression: Executed via the glmnet R package with 10-fold cross-validation to minimize overfitting. The optimal lambda was selected based on the minimum binomial deviance criterion.

Random Forest: Applied using the randomForest package with 1,000 trees and Gini impurity index for feature importance ranking.

Support Vector Machine (SVM): Implemented via the e1071 package with a radial basis function (RBF) kernel. Hyperparameters were tuned through grid search on 5-fold cross-validation.

Feature subsets identified by all three algorithms were intersected to construct a consensus diagnostic panel. Model performance was evaluated using ROC curves and precision-recall metrics on held-out validation cohorts.

### 2.2 Functional and pathway enrichment analysis

Gene Ontology (GO), Kyoto Encyclopedia of Genes and Genomes (KEGG), and Gene Set Enrichment Analysis (GSEA) were conducted using clusterProfiler (v4.8.0) and fgsea (v1.22.0). Significant terms were defined as FDR < 0.05 for GO/KEGG and normalized enrichment score (NES) > |1.6| for GSEA. Immune cell infiltration was estimated using CIBERSORT and xCell, and Spearman’s correlation was used to assess the relationship between S100A11 expression and immune cell subsets.

### 2.4 Diagnostic model construction

A diagnostic panel comprising five genes (S100A11, CA4, PDCD4, GSTM2, SORD) was refined via LASSO regression. Model performance was evaluated through 10-fold cross-validation, with receiver operating characteristic (ROC) curves and precision-recall analysis.

### 2.5 Molecular docking

The molecular docking study was performed using the Cavity-detection guided Blind Docking webserver. The protein structure of S100A11 was retrieved from the Protein Data Bank (PDB ID: 1v4z). The ligand, Diallyl trisulfide, was prepared in 3D conformer format (sdf file) using the online tool Conformer3D. The docking process was guided by cavity detection, and the top 5 detected CurPockets were analyzed. The docking parameters and scoring functions were set to default values provided by the webserver.

## 3 *In vitro* and *vivo* experiments

### 3.1 Cell culture and ethical sample collection

Colorectal cancer cell lines HCT116 and SW480 (Shanghai Institute of Cell Biology) were maintained at 37°C in a humidified atmosphere with 5% CO_2_, using DMEM (Biosharp) supplemented with 10% fetal bovine serum (FBS, Tianhang), 100 U/mL penicillin, and 100 µg/mL streptomycin. Matched tumor and adjacent normal tissues were obtained from 10 treatment-naïve CRC patients at Hangzhou Third People’s Hospital following ethical approval and informed consent.

### 3.2 siRNA-mediated S100A11 knockdown

S100A11 siRNA and scrambled siRNA (si-NC) were synthesized by Gene Pharma. Transfection was performed using RFect (Baidai,China) following the manufacturer’s protocol. Knockdown efficiency was validated by qPCR.

### 3.3 qPCR analysis

RNA Extraction and Reverse Transcription: Total RNA was extracted with Tissue RNA Purification Kit Plus (Yishan Biotechnology, China), Total RNA (1.0 μg) was reverse-transcribed using Evo M-MLV RT Premix (Accurate Biotechnology) followed by TPX2 mRNA quantification with SYBR Green Premix Pro Taq HS (Accurate Biotechnology) on a Bio-Rad real-time PCR system, using GAPDH as the endogenous control. The 2^−ΔΔCt method was used to calculate relative expression. All experiments were conducted in triplicate, and primer sequences are listed in **Supplementary Table S1**.

### 3.4 Immunohistochemistry

Paraffin-embedded CRC and adjacent normal tissues were deparaffinized, antigen-retrieved, and incubated with anti-S100A11 antibody (1:100, Proteintech,China). Sections were incubated with secondary antibody (15 min), followed by DAB development, hematoxylin counterstaining, dehydration and mounting, then visualized and imaged using a Leica DM6B microscope.

### 3.8 CCK-8 assay

Transfected cells were seeded in 96-well plates (3×10³ cells/well) and cultured for 24–96 h post-seeding. CCK-8 reagent (10 μL/well; Biosharp) was added at indicated timepoints, incubated for 2 h, then absorbance at 450 nm was quantified using a BioTek microplate reader. Experiments included six technical replicates per sample and were performed in triplicate.

### 3.6 Colony formation assay

CRC cells (800/well) were cultured in 6-well plates with DMEM (10% FBS) for 14 days at 37°C/5% CO_2_, with medium refreshed every 3 days. Colonies were fixed with 4% methanol (15 min, RT), stained with 0.5% crystal violet (20 min), washed, air-dried, and quantified (≥50 cells/colony) using ImageJ. Experiments included triplicate wells per condition and three biological replicates.

### 3.7 Apoptosis assay

Apoptosis was assessed using the FITC Annexin V Detection Kit (BD Biosciences). Cells were harvested, resuspended, and sequentially stained with Annexin V-FITC (15 min, room temperature, light-protected) followed by PI counterstaining. Analysis was performed using a Beckman Coulter CytoFlex flow cytometer.

### 3.8 Western blot analysis

Proteins were extracted, quantified, and separated on 12% SDS-PAGE before PVDF transfer. Membranes were blocked, incubated with primary and secondary antibodies, and signals detected via ECL.

### 3.9 Animal experiment

BALB/c nude mice (4-week-old males, n=8) were randomized into control and si-S100A11 groups (n=4 each). Subcutaneous injections of 2×10⁶ cells in 200 μl PBS/Matrigel (1:1) were administered in the right flank. Tumor growth was monitored for 28 days before euthanasia, followed by tumor excision, weight measurement, and photographic documentation. Protocols were approved by the Third People’s Hospital of Hangzhou Animal Ethics Committee.

## 4 Statistical analysis

Bioinformatics: FDR-adjusted p < 0.05 was considered significant.

Data are presented as mean ± SD. Comparisons used Student’s t-test (two groups) or ANOVA (multiple groups). p < 0.05 was deemed statistically significant. Analyses were conducted using GraphPad Prism 8.0.

## 5 Results

### 5.1 Identification of differentially expressed genes

**Figure 1:**
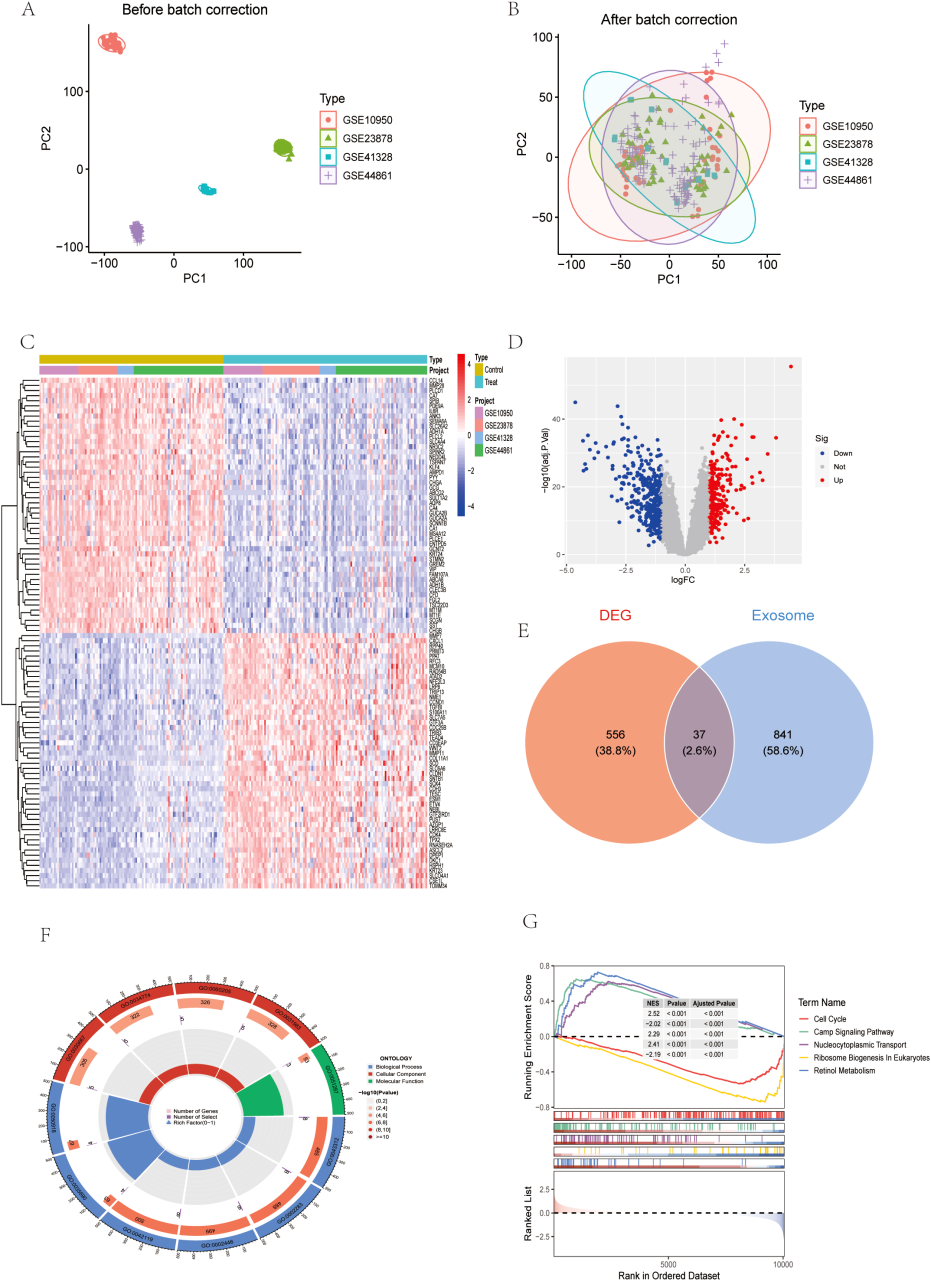
Identification of differentially expressed genes (DEGs). **(A, B)** GSE dataset before and after batch correction. **(C)** Heatmap of the dataset for the batch **(D)** Volcano maps for this batch of datasets. **(E)** Wayne diagram of DEG and exosome genes. **(F)** Circular chart of GO analysis results. **(G)** GSEA analysis results.

To identify colon cancer (CC)-related DEGs, we conducted a bioinformatics analysis using data from GEO and TCGA. Specifically, we analyzed gene expression profiles from GSE10950, GSE23878, GSE41328, and GSE44861 in GEO, comparing CC and normal colon tissues. After batch correction (**Figures 1A, B**), a total of 593 DEGs were identified, and 239 genes were up-regulated and 354 genes were down-regulated in CC tissues compared with normal tissues (**Figure 1D**). The heatmap further visualized the important DEGs, highlighting their expression patterns in different samples (**Figure 1C**). Exosome-related DEGs in the batch of dataset were screened by GeneCard database (**Figure 1E**).

### 5.2 Screening of exosome-related genes

**Figure 2:**
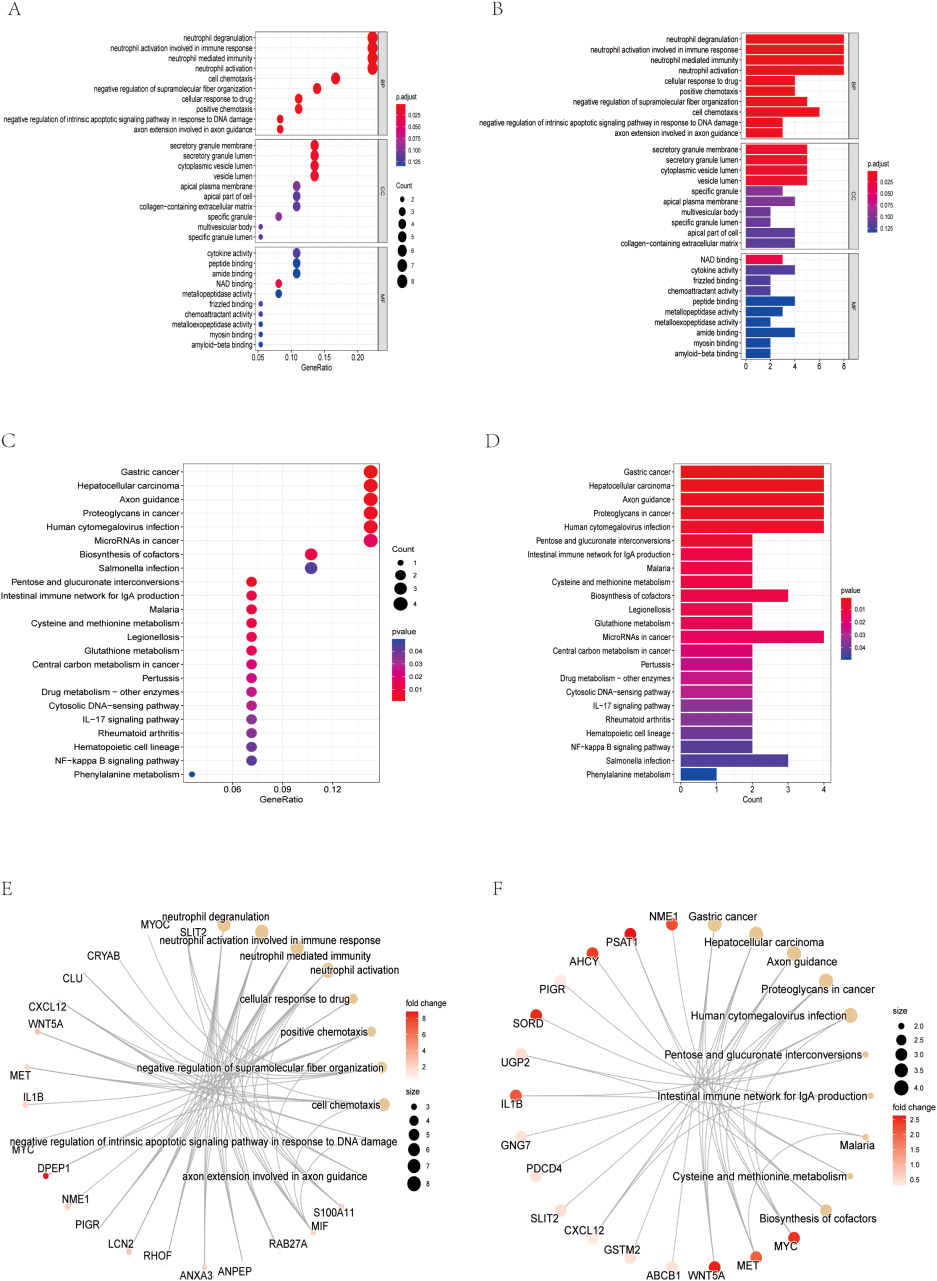
Screening of exosome-related genes. **(A)** Bubble chart of GO analysis results. **(B)** Bar chart of GO analysis results **(C)** Bubble chart of KEGG analysis results **(D)** Bar chart of KEGG analysis results. **(E)** Network diagram of the GO analysis results. **(F)** Network diagram of the KEGG analysis results.

Considering the critical role of exosomes in intercellular communication and the potential impact on CRC mechanisms, we focused on exosome-related genes in the identified DEGs. Subsequently, GO, KEGG and gene set enrichment analysis (GSEA) were performed on the differential exosome genes, and the GO results showed that they play an important role in neutrophil degranulation, neutrophil activation involved in immune response, neutrophil mediated immunity and neutrophil activation regulation, among other biological processes (**Figures 1F**, **2A, B, E**). Integrated KEGG pathway analysis of differentially expressed exosomal genes in colorectal cancer highlighted significant enrichment of pathways associated with tumor progression, immune regulation and metabolic reprogramming. Key cancer-related pathways such as “Gastric cancer”, “Hepatocellular carcinoma” and “Proteoglycans in cancer” were prominently identified in network, bar and bubble plots, and genes such as NME1, PSAT1, AHCY and PIGR were significantly involved. Metabolic pathways such as ‘cysteine and methionine metabolism’, ‘cofactor biosynthesis’ and ‘central carbon metabolism in cancer’ were also enriched, highlighting altered metabolic dynamics in CRC-derived exosomes. Immune-related pathways, such as the “intestinal IgA-producing immune network” and the “IL-17 signaling pathway”, were key contributors, and genes such as IL1B, GNG7, and PDCD4 may mediate immune evasion or inflammatory responses. In addition, infection-related pathways (“human cytomegalovirus infection”, “Salmonella infection”) and stress-responsive pathways (“glutathione metabolism”) suggest a role for exosomes in microenvironmental adaptation (**Figures 2C, D, F**). The consistency of these findings in visualization methods (network diagrams, bar charts, and bubble charts) strengthens their biological relevance and suggests that exosomal genes are involved in the pathogenesis of CRC through different mechanisms. GeneSetEnrichmentAnalysis (GSEA) of colorectal cancer exosomal genes showed significant enrichment of pathways associated with cell proliferation and metabolic regulation. The most highly enriched gene sets included cell cycle (NES=2.52), nucleocytoplasmic transport (NES=2.29), and ribosome biogenesis in eukaryotes (NES=2.41), suggesting upregulation of oncogenic processes. In contrast, cAMP signaling pathway (NES=-2.02) and retinol metabolism (NES=-2.19) appeared to be suppressed, suggesting dysregulation of metabolic homeostasis (**Figure 1G**). These results emphasize the dual role of exosomal genes in promoting tumorigenesis and impairing cell maintenance mechanisms in colorectal cancer.

### 5.3 Machine learning identifies robust exosomal biomarkers for CRC diagnosis

**Figure 3:**
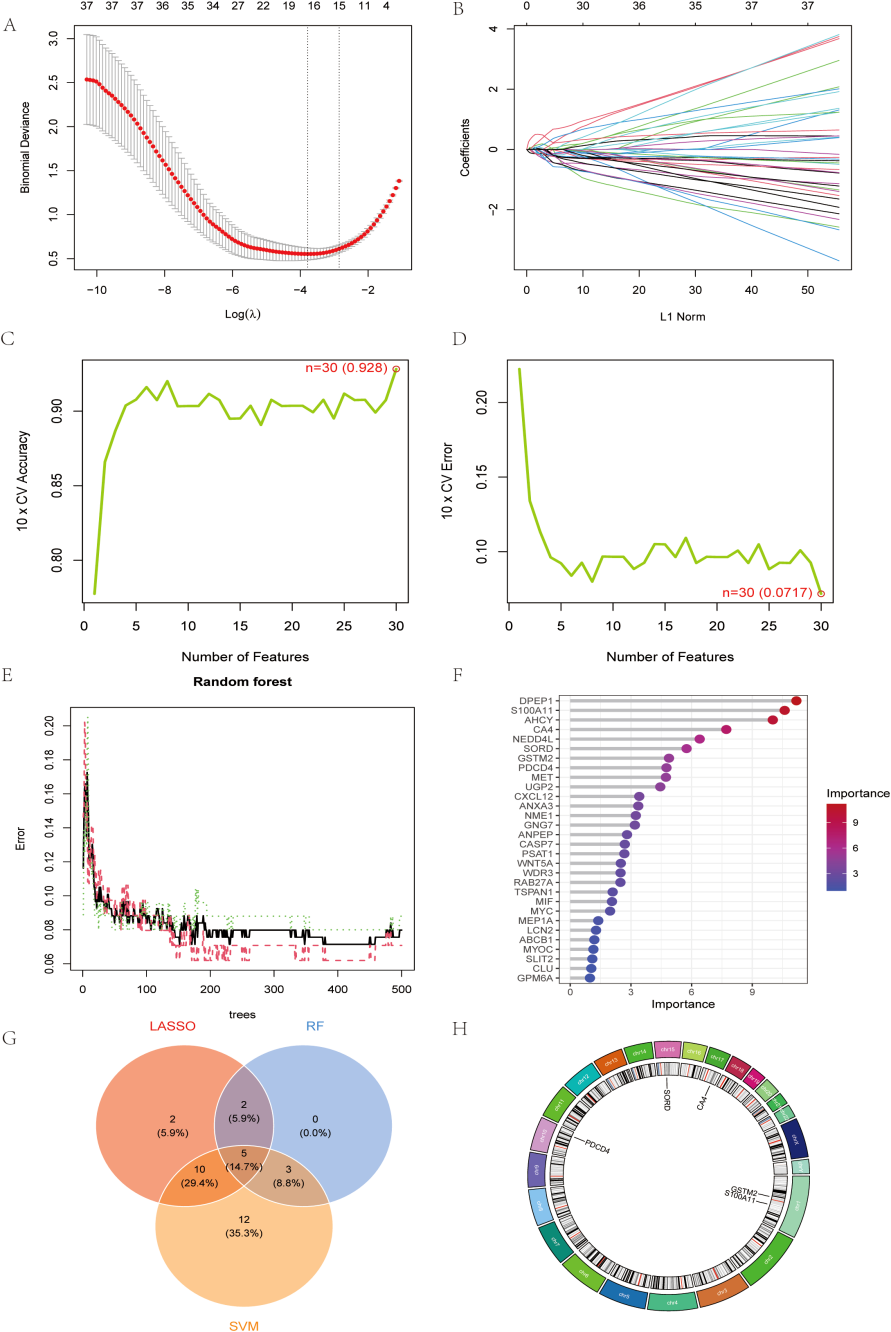
Machine learning identifies robust exosomal biomarkers for CRC diagnosis **(A)** LASSO regression cross-validation plot. **(B)** Path diagram of LASSO regression coefficients **(C)** Plot of number of features against cross-validation accuracy **(D)** Plot of number of features vs. cross-validation error rate. **(E)** Plot of error rate vs. number of trees for random forest models. **(F)** Gene Importance Ranking Chart. **(G)** Wayne result plots for LASSO, RF and SVM. **(H)** Circular genome visualization map.

To further narrow down the candidate genes, we employed LASSO regression (**Figures 3A, B**), SVM (**Figures 3C, D**) and RF (**Figures 3E, F**) algorithms to critically analyze the characterization selection and validation of exosomal biodiagnostic markers for colon cancer.LASSO regression identified 30 key genes with coefficients stabilized at the optimal log(λ) values of -2.0 to -4.0 that minimized binomial bias. Ten-fold cross-validation demonstrated the robust performance of the model with an accuracy of 0.928 and an error rate of 0.072. random forest analysis ranked DPEP1, S100A11, AHCY, and CA4 as the top contributors to the predictive power, with significance scores of more than 6.0. the intersection of the genes screened by these methods identified a robust set of candidate genes (**Figure 3G**), CA4, S100A11, PDCD4, GSTM2, and SORD for subsequent experimental validation. Chromosome mapping revealed that the selected genes were enriched on chromosomes 1, 3, 15, and 17, suggesting potential genomic hotspots for exosome regulation associated with CRC (**Figure 3H**).

**Figure 4:**
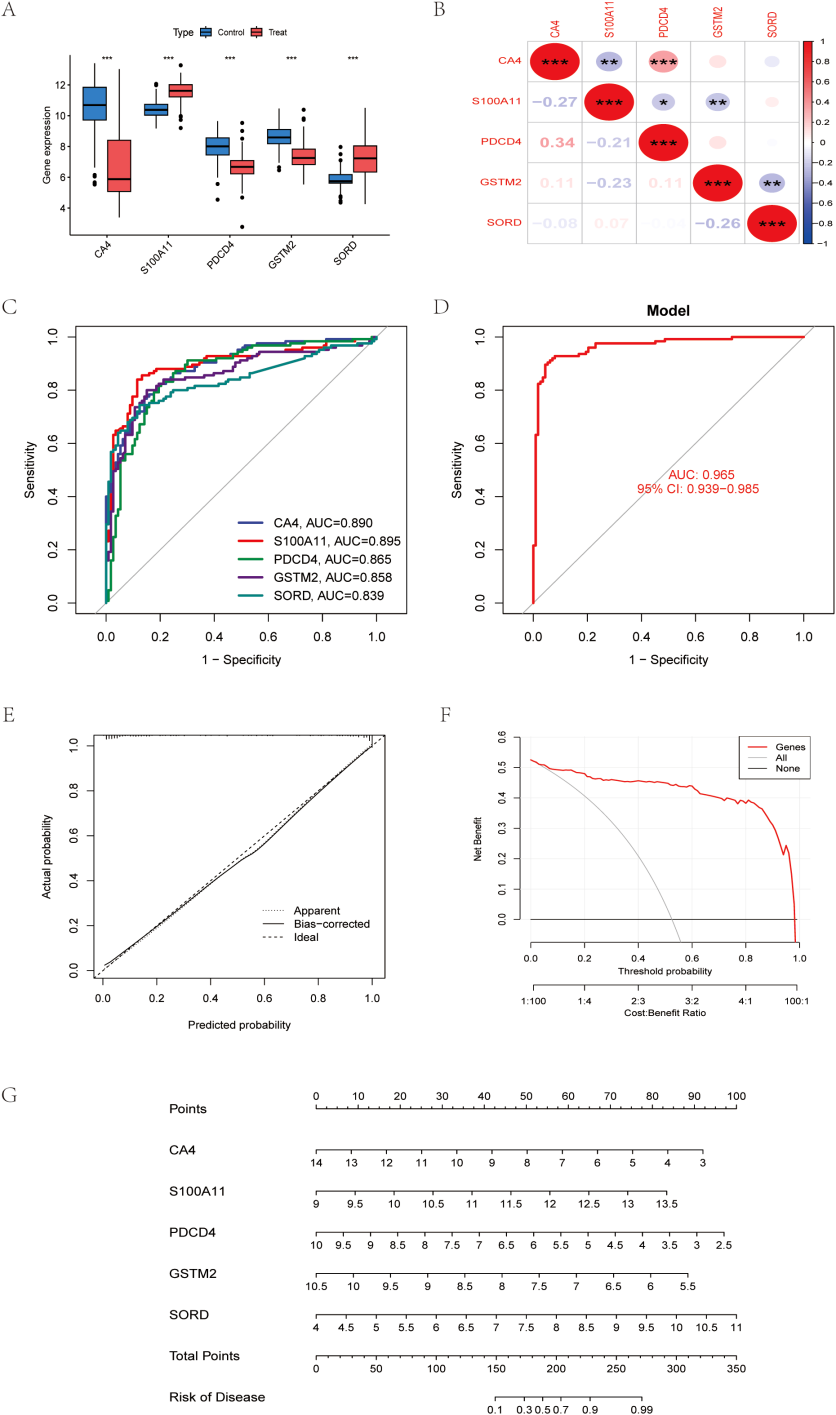
Machine learning identifies robust exosomal biomarkers for CRC diagnosis **(A)** Differential expression maps of five reliable exosomal genes. **(B)** Correlation between five reliable exosome genes **(C)** Receiver operating characteristic (ROC) curves for genes **(D)** Receiver Operating Characteristics (ROC) curves for models. **(E)** Calibration curve of the model. **(F)** Decision Curve Analysis, (DCA). **(G)** Column line graph constructed from CA4, S100A11, PDCD4, GSTM2, SORD. *P < 0.05, **P < 0.01, ***P < 0.001.

The performance of this diagnostic model was evaluated by cross-validation with an AUC of 0.965 for LASSO regression (**Figures 4C, D**). We further analyzed the expression of the selected genes in colon cancer and the correlation analysis between each (**Figures 4A, B**). Decision curve analysis (DCA) showed that compared with the “all” or “none” strategy, the exosomal gene-based model provided superior net benefits at various threshold probabilities (10%-90%), especially when the cost-benefit ratio favored early detection of CRC (**Figure 4F**). Nomograms integrating CA4, S100A11, PDCD4, GSTM2, and SORD enabled quantitative risk stratification, with total scores (0–350) correlating with increasing probability of disease (0.1-0.99) (**Figure 4G**). Calibration analysis showed that the predicted probabilities were in excellent agreement with the actual probabilities (bias correction curves were close to the ideal values), confirming the reliability of the model (**Figure 4E**). These results validate the clinical utility of exosomal biomarkers in predicting CRC risk.

### 5.4 Exosomal biomarkers modulate immune microenvironment and therapeutic response

**Figure 5:**
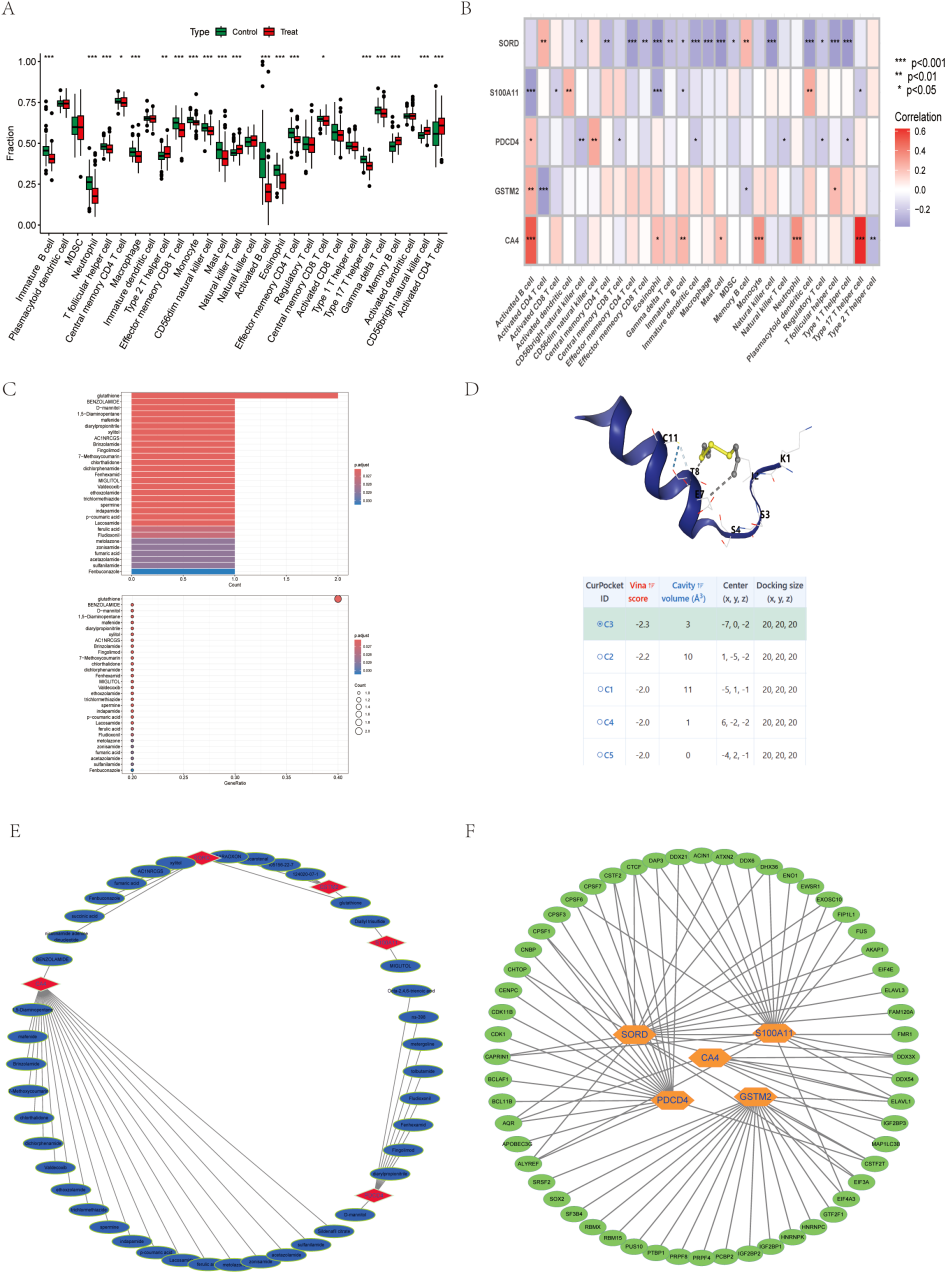
Exosomal Biomarkers Modulate Immune Microenvironment and Therapeutic Response **(A)** Relationship of the batch of datasets to immune infiltration. **(B)** Association of CA4, S100A11, PDCD4, GSTM2, SORD and immune infiltration **(C)** Drug enrichment analysis **(D)** Molecular docking of S100A11. **(E)** Drug regulatory networks. **(F)** Regulatory networks of RNA-binding proteins. *P < 0.05, **P < 0.01, ***P < 0.001.

Comparative analysis of immune cell infiltration in colorectal cancer (CRC) tissues and adjacent normal tissues revealed that the tumor microenvironment was skewed toward immunosuppression, with CRC tissues displaying a decrease in effector memeory CD8+ T cells and macrophages (**Figure 5A**). Five exosomal genes (S100A11, PDCD4, GSTM2, SORD and CA4) showed different immune correlations: while S100A11 was strongly associated with dendritic cell infiltration, GSTM2 was inversely associated with activated CD4 T cell, which suggests their immune-regulatory role (**Figure 5B**). Drug enrichment analysis and the pharmacogenomics network identified S100A11 as a sensitivity marker for Diallyl trisulfide and MIGLITOL, highlighting therapeutic vulnerability (**Figures 5C, E**). Molecular docking confirmed high affinity binding between S100A11 and diallyl trisulfide, supporting its potential as a drug target (**Figure 5D**). RNA-binding protein networks reveal key regulators of diagnostic markers such as S100A11 and PDCD4., suggesting a role for post-transcriptional control mechanisms in CRC progression (**Figure 5F**). Together, these findings position exosomal biomarkers as key players in CRC immune evasion, drug response, and targeted therapy.

### 5.5 S100A11 expression and functional role in colorectal cancer

**Figure 6:**
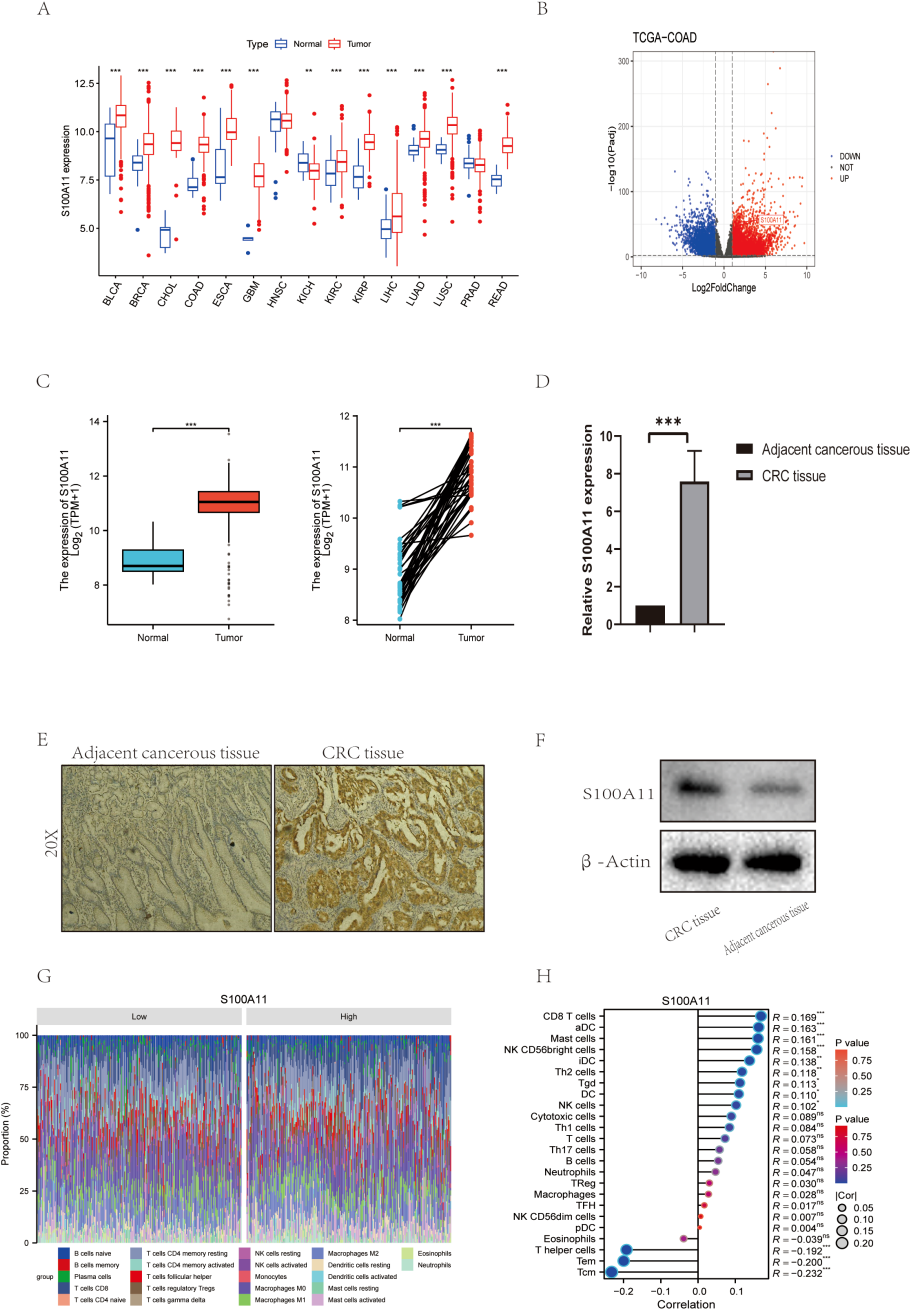
S100A11 Expression and Functional Role in Colorectal Cancer **(A)** Expression of S100A11 in a variety of cancers. **(B)** Volcano diagram of S100A11 in TCGA-COAD. **(C)**Expression of S100A11 in TCGA-COAD (unpaired and paired samples) **(D)** S100A11 expression in 10 pairs of colon cancer samples. **(E)** Immunohistochemical results of S100A11 in patients with colon cancer. **(F)** Western blot results of S100A11 in colon cancer patients. **(G)** Stacked Bar Chart of S100A11 immune infiltration profiles **(H)** Correlation analysis of S100A11 with immune infiltration *P < 0.05, **P < 0.01, ***P < 0.001. ns: not significant, indicating P > 0.05.

We performed a comprehensive analysis of the expression profile of S100A11 using bioinformatics and experimental methods. Pan-cancer analysis was first performed and revealed that S100A11 was differentially expressed in a variety of malignant tumor tissues (**Figure 6A**), subsequent bioinformatics analysis using our TCGA-COAD dataset showed that S100A11 levels were notably increased in colon cancer tissues compared to normal tissues. (**Figures 6B, C**). qRT-PCR analysis showed that compared to neighboring normal tissues, the tumor tissue the S100A11 mRNA level was increased 3.2-fold (p<0.001, **Figure 6D**). Similarly, IHC staining showed a high amount of S100A11 protein expression in colon cancer tissues, while minimal staining was observed in adjacent normal tissues (**Figure 6E**). The Western blot results showed the same trend (**Figure 6F**). In summary, these findings suggest that S100A11 is highly expressed in colon cancer and may play an important role in colon cancer progression.

In addition, the relationship between S100A11 expression and immune cell infiltration was analyzed. The results showed that S100A11 expression was significantly negatively correlated with the infiltration of several immune cell types, including T helper cells, Tem and Tcm (**Figure 6H**). This suggests that S100A11 may regulate the tumor immune microenvironment by affecting the infiltration and activity of these immune cells. In addition, analysis of the proportion of immune cell subpopulations showed that S100A11 expression was associated with a higher proportion of neutrophils and a lower proportion of regulatory T cells (**Figure 6G**), suggesting that S100A11 may affect the balance of immune cell subpopulations in the tumor microenvironment, which may lead to immune evasion by colon cancer cells.

In conclusion, our study reveals that S100A11 is elevated in colon cancer tissues and correlates with immune cell infiltration and activity, suggesting its potential as a biomarker and target for treatment in colon cancer.

### 5.6 S100A11 depletion suppresses colorectal cancer progression *in vitro* and *in vivo*

**Figure 7:**
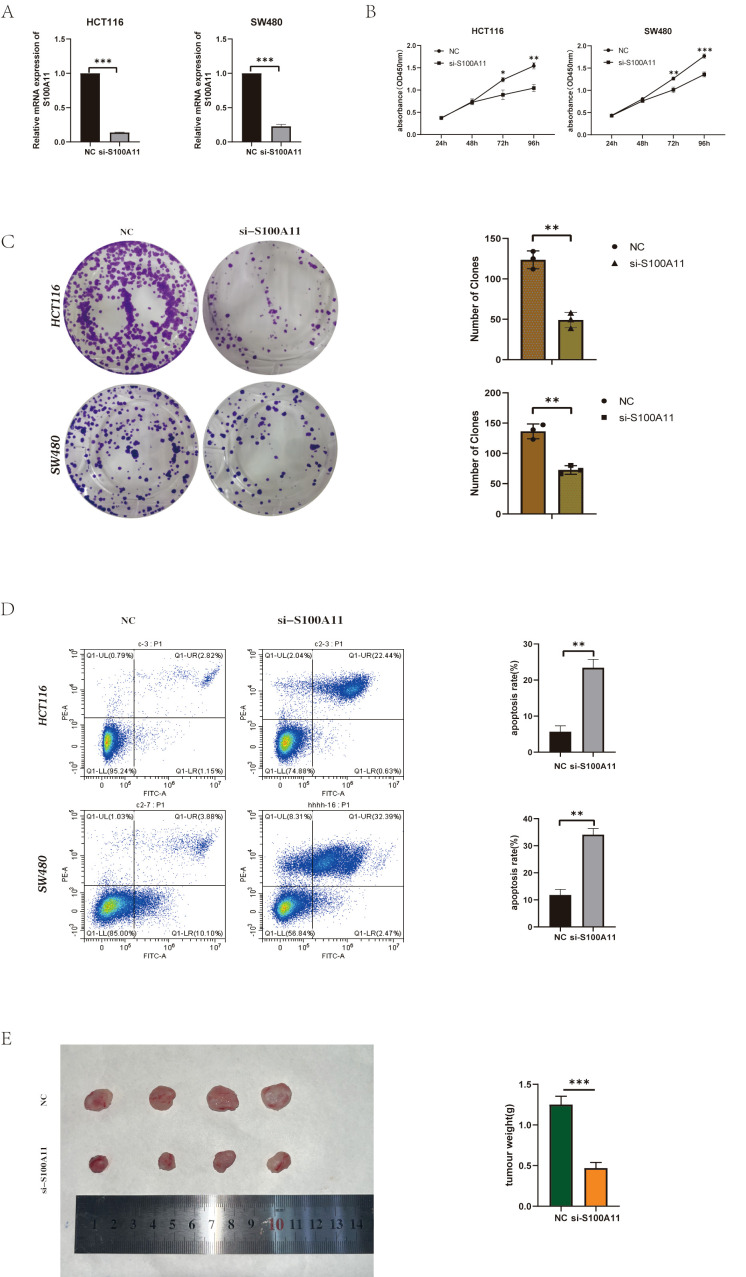
S100A11 depletion suppresses colorectal cancer progression *in vitro* and *in vivo* **(A)** Knockdown efficiency of S100A11 in two cell lines. **(B)** CCK-8 results for two cell lines **(C)** Results of plate cloning experiments with two cell lines **(D)** Results of apoptosis experiments in two cell lines. **(E)** Tumor formation in nude mice. *P < 0.05, **P < 0.01, ***P < 0.001.

To explore the functional role of S100A11 in colorectal cancer progression, Researcher performed siRNA-mediated knockdown. Silencing of S100A11 significantly inhibited cell proliferation compared with the negative control, as evidenced by reduced cell viability in the CCK-8 assay (**Figure 7B**). Plate cloning experiments also demonstrated that toning down the expression of S100A11 inhibited cell proliferation(**Figure 7C**). In contrast, flow cytometry showed a greater than 3-fold increase in apoptosis in S100A11-deficient cells. (**Figure 7D**). Collectively, these results suggest that S100A11 is critical for maintaining the proliferative and anti-apoptotic properties of colorectal cancer cells, emphasizing its potential as a therapeutic target.

To validate these results *in vivo*, we established a xenograft tumor model in nude mice. Tumor growth generated by S100A11-knockout cells was significantly attenuated compared to controls, (**Figure 7E**). These results indicate that S100A11 depletion inhibited cell proliferation and survival *in vitro*, and markedly reduced tumor growth *in vivo*. Collectively, these data emphasize that S100A11 is a key oncogenic driver of CRC with the dual role of promoting malignant progression and evading therapeutic intervention.

## 6 Discussion

In this study, we systematically revealed the dual regulatory roles of exosome-related genes in colorectal cancer by integrating multi-omics data and experimental validation - both as high-precision diagnostic markers and involved in the remodeling of the tumor immune microenvironment and the regulation of chemotherapeutic resistance. Among them, the machine learning-driven five-gene diagnostic model (S100A11/CA4/PDCD4/GSTM2/SORD) demonstrated performance beyond the existing single-marker detection system in cross-cohort validation, with an AUC value of 0.965, which provides a new paradigm for the non-invasive diagnosis of early colorectal cancer. More importantly, we found that several members of this gene combination (CA4, SORD) are closely related to tumor metabolic reprogramming, suggesting that exosomes could influence nutrient sensing and drug response of tumor cells by regulating the cystine-methionine metabolic axis, a finding that provides a theoretical basis for the development of metabolism-intervention-based combination therapy strategies.

The functional analysis of the core molecule S100A11 is an important breakthrough in our research. The role of S100A11 in colorectal cancer remains debated. Previous studies suggest that desmethyl esters regulate the NF-κB signaling pathway, leading to S100A11 inhibition (36), and there is also evidence to support the possibility that LBX2-AS1 may promote proliferation and invasion by regulating S100A11 (37). In this study, we observed a strong association between high S100A11 expression and immunosuppressive microenvironmental traits, and that knockdown of S100A11 inhibited tumor cell proliferation and enhanced apoptosis. This dual “pro-cancer” property may be related to its calcium-dependent conformational changes (38). Molecular docking revealed that S100A11 binds to diallyl trisulfide (DATS), an organosulfur compound that possesses a variety of biological activities including antioxidant, anti-inflammatory, and anticancer effects, which include modulation of a variety of cancer markers among other roles, and modulates a number of hallmark cancer pathways (39), including regulation of the cell cycle, apoptosis, angiogenesis and metastasis (40, 41). Research has demonstrated that DATS can inhibits cancer cell progression at different cell cycle stages, which provides a structural basis for the development of small molecule inhibitors targeting its dimerisation interface.

From a translational medicine perspective, the findings of this study have triple clinical significance: first, the association of CA4 with vascular normalization in diagnostic models suggests that it could be a potential predictor of anti-VEGF therapy efficacy; second, GSTM2-mediated oxaliplatin resistance can be reversed by inhibition of glutathione metabolism pathway, which provides a new idea for overcoming chemo-resistance; and lastly, the post-transcriptional regulation of PDCD4 mechanism involves RNA-binding proteins such as ALYREF/CDK1, suggesting that targeting the mRNA stability regulatory network may enhance the efficacy of existing targeted therapies. However, the clinical application of exosomal gene markers still faces challenges: on the one hand, the heterogeneity of peripheral blood exosomes may lead to fluctuations in marker abundance; on the other hand, the physiological functions of S100A11 in normal tissues require that targeted therapies must be equipped with precise tumor-specific delivery systems.

Despite these strengths, we recognize several limitations. Most notably, while we integrated multiple datasets and applied 10-fold cross-validation to reduce overfitting risk, we did not conduct an independent external validation due to the unavailability of suitable datasets. Future work will focus on prospective validation using multi-center clinical cohorts to assess the reproducibility and real-world applicability of the model.

The methodological innovations of this study lie in minimizing overfitting risk through LASSO-SVM-Random Forest triple algorithm cross-validation, addressing deviations in bioinformatics predictions by combining molecular docking with functional experiments, and enhancing tumor microenvironment analysis through the integration of xCell and CIBERSORT dual deconvolution algorithms.

However, the conclusions are based on retrospective data and xenograft models, which do not fully replicate the dynamic interactions between the human immune system and tumors. Additionally, further validation of the correlation between exosomal mRNA and the proteome is needed, using techniques like mass spectrometry.

Future studies should explore the following aspects: establishing a prospective multicenter cohort to assess the clinical translational value of the diagnostic model; developing a nanocarrier system based on S100A11 targeting to achieve controlled release at the tumor site by utilizing its calcium-responsive properties; and resolving the spatiotemporal regulatory mechanism of exosome CA4 in the formation of pre-metastatic microenvironment. These explorations will promote the leap of exosome markers from basic research to clinical practice, and ultimately achieve early precision intervention and personalized treatment of colorectal cancer.

## 7 Conclusion

This study comprehensively elucidates the critical roles of exosome-related genes in colorectal cancer (CRC) pathogenesis, diagnosis, and therapeutic intervention. By integrating bioinformatics and machine learning approaches, we identified 593 differentially expressed genes (DEGs) in CRC, with exosome-related genes prominently enriched in pathways driving tumor progression (gastric cancer, hepatocellular carcinoma), immunosuppression (IL-17/NF-κB signaling), and metabolic reprogramming (cysteine and methionine metabolism). A robust diagnostic panel (S100A11/CA4/PDCD4/GSTM2/SORD), validated through LASSO, SVM, and random forest algorithms, demonstrated high accuracy (AUC=0.965) and clinical utility for early CRC detection. Functional characterization revealed that S100A11, a key exosomal biomarker, promotes CRC progression *in vitro* by enhancing proliferation, and anti-apoptotic signaling, while its *in vivo* knockdown suppressed tumor growth, underscoring its oncogenic potency. Furthermore, S100A11 expression correlated with an immunosuppressive microenvironment marked by reduced immune cells, implicating its role in immune evasion. Pharmacogenomic analyses linked exosomal genes to drug sensitivity and resistance, highlighting their therapeutic relevance. These findings position exosome-derived biomarkers, particularly S100A11, as pivotal drivers of CRC malignancy and promising targets for precision oncology. Future studies should validate these biomarkers in prospective clinical cohorts and explore mechanisms underlying exosome-mediated immunomodulation and metabolic reprogramming. Collectively, this work lays the foundation for developing exosome-based diagnostic tools and targeted therapies to improve outcomes for CRC patients.

## Data Availability

The datasets presented in this study can be found in online repositories. The names of the repository/repositories and accession number(s) can be found below: TCGA-COAD dataset: https://www.cancer.gov/tcga and GEO datasets (GSE10950: https://www.ncbi.nlm.nih.gov/geo/query/acc.cgi?acc=GSE10950; GSE23878: https://www.ncbi.nlm.nih.gov/geo/query/acc.cgi?acc=GSE23878; GSE41328: https://www.ncbi.nlm.nih.gov/geo/query/acc.cgi?acc=GSE41328; GSE44861: https://www.ncbi.nlm.nih.gov/geo/query/acc.cgi?acc=GSE44861).
