# Supplementary figures and images for: S100A11 as an immune-related exosomal driver of colorectal cancer progression: a novel diagnostic biomarker
Source: Front Oncol. 2025 Jun 11;15:1590128. doi: 10.3389/fonc.2025.1590128 (PMC12187594; doi:10.3389/fonc.2025.1590128)

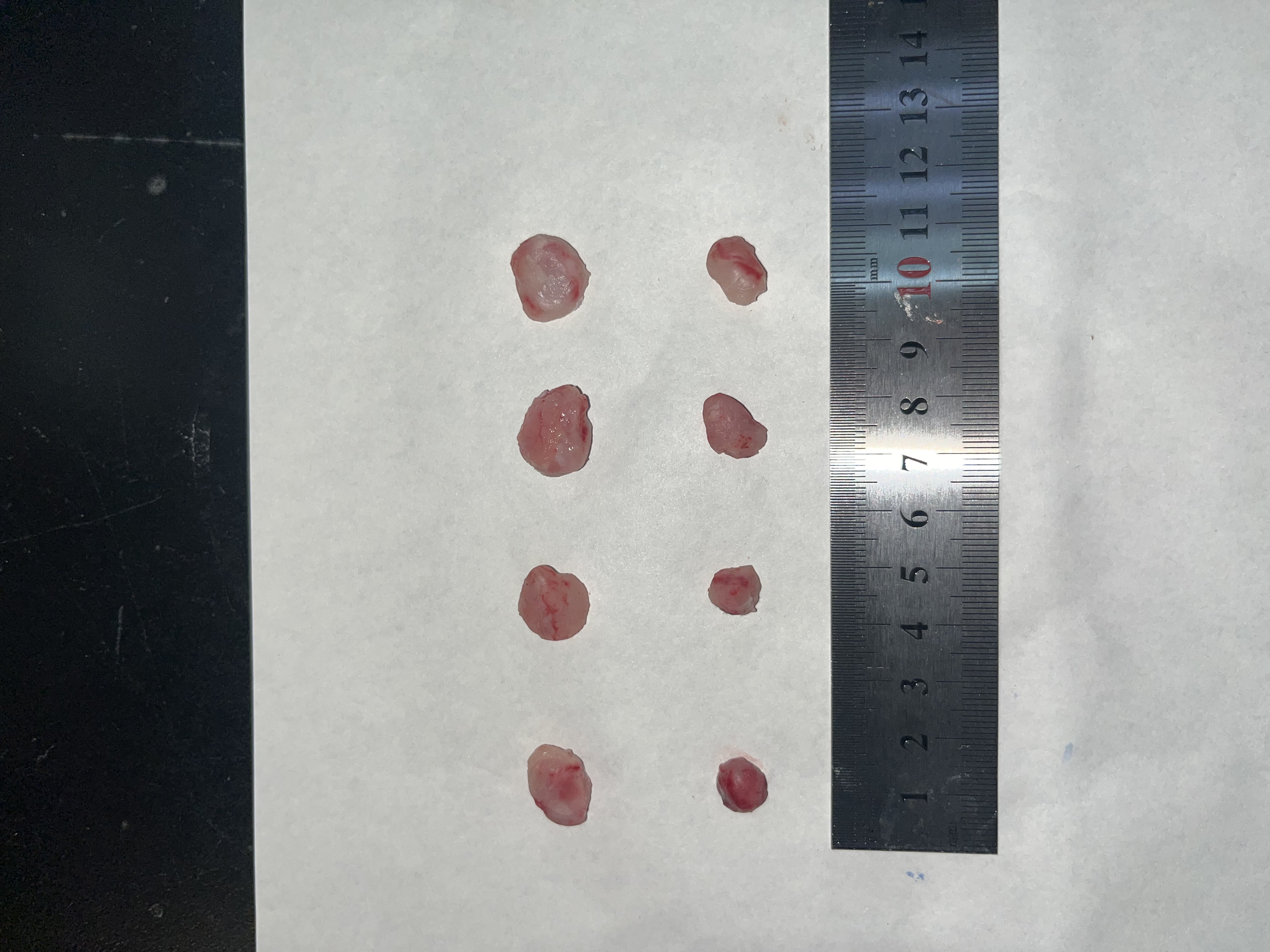

Supplement: Supplementary file 1 [file Image1.jpeg]
