# Supplementary material for: S100A11 as an immune-related exosomal driver of colorectal cancer progression: a novel diagnostic biomarker
Source: Front Oncol. 2025 Jun 11;15:1590128. doi: 10.3389/fonc.2025.1590128 (PMC12187594; doi:10.3389/fonc.2025.1590128)
Supplement: Supplementary file 2 [file Table1.docx]

**Table.S1** Interfering sequences of S100A11 used for transfection

| **Genes** | **Sequences** |
| --- | --- |
| S100A11  GAPDH  si-S100A11  Si-NC | Sense:5’- TGGCTTGCCATGACTCCTTC-3’  Antisense:5’- GGAAAGGGGGTGGGTTTGAA-3’  Sense:5’-CCACTCCTCCACCTTTG-3’  Antisense:5’-CACCACCCTGTTGCTGT-3’  Sense:5’- CUGGAAAGGAUGGUUAUAATT-3’  Antisense:5’- UUAUAACCAUCCUUUCCAGTT-3’  Sense:5’- UUCUCCGAACGUGUCACGUdTdT-3’  Antisense:5’- ACGUGACACGUUCGGAGAAdTdT-3’ |
